# Fallbericht – eine Hornhautspenderin mit einem positiven SARS-CoV-2-Nachweis

**DOI:** 10.1007/s00347-020-01264-6

**Published:** 2020-11-09

**Authors:** D. Wille, J. Heinzelmann, N. Hofmann, M. Börgel, A. Kehlen, A. Müller, A. Viestenz

**Affiliations:** 1 Mitteldeutsche Corneabank Halle, Universitätsklinik und Poliklinik für Augenheilkunde, Universitätsklinikum Halle, Martin-Luther-Universität Halle, Halle (Saale), Deutschland; 2 Deutsche Gesellschaft für Gewebetransplantation gGmbH, Hannover, Deutschland; 3 Institut für Medizinische Mikrobiologie, Universitätsklinikum Halle, Martin-Luther-Universität Halle, Halle (Saale), Deutschland

## Hintergrund

Die Augenhornhautspende unterliegt gesetzlichen Regelungen, die die Sicherheit und Qualität des Gewebes sicherstellen und dem Empfängerschutz dienen. Bei neu auftretenden Krankheitserregern muss die Spenderprüfung angepasst werden.

Am 31.12.2019 wurde die *World Health Organisation* (WHO) über Fälle von Pneumonien unbekannter Ätiologie (China) informiert. Das verursachende Virus wurde als „severe acute respiratory syndrome coronavirus 2“ (SARS-CoV-2) bezeichnet. Am 11.03.2020 erfolgte die Einstufung als eine Pandemie durch die WHO.

Die deutsche Bundesoberbehörde für Gewebeeinrichtungen, das Paul-Ehrlich-Institut, fordert präventive Maßnahmen bei der Prüfung der Eignung möglicher Spender. Diese beziehen sich auf bestätigte Kontakte mit SARS-CoV-2-infizierten Personen und zurückliegende bestätigte SARS-CoV-2-Infektionen [1]. Eine erweiterte Spenderprüfung liegt im Ermessen der Gewebeeinrichtungen. Alle Spenden der Deutschen Gesellschaft für Gewebetransplantation gGmbH (DGFG) werden entsprechend einer strikten Risikobewertung unterzogen.

Derzeit ist die Gefahr einer SARS-CoV-2-Übertragung durch Gewebetransplantationen nicht bekannt.

## Falldarstellung und Testmethoden

Eine 70-jährige Frau wurde durch ein kooperierendes Krankenhaus als potenzielle Gewebespenderin gemeldet. Die Todesursache war eine Intoxikation. Die medizinische und soziale Anamneseerhebung verliefen unauffällig, ohne Hinweise auf Lungenentzündung oder andere Infektionen. Standardmäßig wurde bei Aufnahme ins Krankenhaus ein Nasopharynxabstrich zur Testung auf SARS-CoV‑2 genommen, dessen Ergebnis aber zum Zeitpunkt der Spende nicht vorlag. Die Entnahme der ophthalmologischen Gewebe sowie des postmortalen Abstriches erfolgte 36 h nach dem Versterben. Nach Abschluss der Spende informierte das Krankenhaus die Gewebeeinrichtung über den positiven SARS-CoV-2-Befund des bei Aufnahme der Spenderin entnommenen Abstriches (Tab. 1).

**Table 1:** 

	Krankenhaus	Korneabank
Probe	Nasopharynxabstrich (trockener Tupfer)	Nasopharynx‑/Konjunktivaabstrich, gepoolt (trockener Tupfer)
Zeitpunkt der Probennahme	Prämortal	Postmortal
Ergebnis	E‑Gen: CT – 37,8 (Doppelt getestet)^a^ RdRp-Gen: CT – 38,9^a^	N‑Gen: negativ^b^ S‑Gen: negativ^b^ E‑Gen: negativ^c^ RdRp-Gen: negativ^c^
Zeitraum: Probennahme bis Test	22 h	19 h
Zeitabstand: Test Krankenhaus/Test Korneabank	45 h	

^a^Test basierend auf dem Protokoll der Charité Berlin

^b^„Anchor SARS-CoV‑2 PCR Kit“ (Anchor Diagnostics GmbH, Hamburg)

^c^„LightMix® Modular SARS-CoV (COVID19)“ (Roche Diagnostics Deutschland GmbH/TIB MOLBIOL GmbH, Berlin)

Der im Rahmen der Spende abgenommene postmortale Abstrich war nach qRT-PCR-Untersuchung negativ. Aufgrund des positiven Resultates des prämortalen Abstriches wurde die qRT-PCR-Analyse der Spenderhornhaut und des verwendeten Kulturmediums veranlasst. Beide Tests waren negativ. Infolgedessen wurde das prämortale Serum der Spenderin auf die SARS-CoV-2-Antikörper-IgA und -IgG mittels „Anti-SARS-CoV-2-Elisa“ (Euroimmun AG, Lübeck) getestet. Dabei wurde SARS-CoV-2-IgA-Antikörper nachgewiesen (Ratio: 2,35; lt. Herstellerangaben ab Ratio 1,1 positiv). IgG-Antikörper wurden nicht detektiert. Mit weiteren Analysen des Spenderserums mittels verschiedener Lateral-Flow-Assays wurden weder SARS-CoV-2-IgM noch SARS-CoV-2-IgG nachgewiesen (Tab. 2).

**Table 2:** 

Krankenhaus			Korneabank		
Probe	Zeitpunkt	Ergebnis	Probe	Zeitpunkt	Ergebnis
Nasopharynxabstrich	Aufnahme	Positiv (schwach)	Nasopharynx‑/Konjunktivaabstrich	45 h nach der Aufnahme	Negativ
Serum	Aufnahme	IgA: positiv^a^ IgG: negativ^a^ (LFA IgG/IgM: negativ)^b^	Korneagewebe und Kulturmedium	9 Tage nach der Aufnahme	Negativ

^a^„Anti-SARS-CoV-2-Elisa“ (Euroimmun AG, Lübeck)

^b^Lateral-Flow-Assays: „mö-screen 2019-NCOV“ (möLab GmbH, Langenfeld), „Rapid 2019-NCOV IgG/IgM Combo Test Card“ (Xiamen Boson Biotech Co. Ltd; Xiamen/China), „COVID-19 IgG/IgM Rapid Test Kit“ (Wuhan UNscience Biotechnology Co. Ltd; Wuhan/China)

Um die abweichenden Testbefunde des Krankenhauses und der Gewebeeinrichtung abzugleichen, wurden die Probennahmebedingungen verglichen. Die Abstriche wurden jeweils mit trockenem Tupfer als Nasopharynxabstrich durchgeführt. Die Lagerzeiten waren vergleichbar. Die Zwischenlagerung erfolgte bei 2–8 °C. Beide Testlabore verwendeten unterschiedliche Testkits. Das vom Krankenhaus beauftragte Labor verwendete einen auf dem Protokoll der Charité Berlin basierenden Test, der die Genomregionen des E‑Gens (*Envelope-Gen*) und RdRp-Gens (*RNA-dependent-RNA-polymerase-Gen*) nachweist. Der qRT-PCR-Test des positiven Nachweises vom Krankenhaus war schwach positiv (CT-Wert zwischen 38 und 40) und damit im Grenzbereich. Der Bestätigungstest aus dem kombinierten Nachweis des E‑Gens (CT-Wert 37,8) und des RdRp-Gens (CT-Wert 38,9) bewegte sich ebenfalls in diesem Bereich. Das Labor der Mitteldeutschen Corneabank Halle (MCH) verwendete das „Anchor SARS-CoV‑2 PCR Kit“ (Anchor Diagnostics, Hamburg). Dieser Test weist das N‑Gen („nucleoprotein“) und das S‑Gen („spike-protein“) nach. Der postmortale Abstrich wurde mit diesem Testsystem negativ auf SARS-CoV‑2 getestet. Um Sensitivitätsunterschiede zwischen den Tests auszuschließen, wurde die Probenrückstellung des postmortalen Abstriches mit dem „LightMix® Modular SARS-CoV (COVID19)“ (Roche Diagnostics/TIB MOLBIOL, Berlin) getestet, wobei auch hier die Genomregionen des E‑Gens und des RdRp-Gens nachgewiesen werden. Mit diesem Test wurde die postmortale Probe ebenfalls negativ auf SARS-CoV‑2 getestet (Tab. 1).

## Diskussion

Im Rahmen der Corona-Pandemie stellt sich die Frage, ob und mit welchen diagnostischen Möglichkeiten die Sicherheit der Hornhauttransplantate erhöht werden kann. Daher wird durch die MCH bei allen Gewebespendern routinemäßig postmortal ein gepoolter Nasopharynx‑/Konjunktivaabstrich mittels qRT-PCR auf SARS-CoV‑2 getestet.

Im vorliegenden Fall ließ das schwach positive PCR-Signal aus dem prämortalen Abstrich vermuten, dass die Infektion der Spenderin mit SARS-CoV‑2 bereits weitestgehend abgeklungen war. Wahrscheinlich hatte die Spenderin keine oder nur leichte Symptome, da sowohl die Anamneseerhebung im Krankenhaus als auch durch die Gewebeeinrichtung unauffällig war. Bedingt durch den im Grenzbereich liegenden CT-Wert des positiven prämortalen Tests ist das negative Ergebnis des postmortalen Tests nicht unerwartet. Die jeweiligen CT-Werte der einzelnen Tests (Tab. 1) entsprechen den in der Literatur beschriebenen Daten [2]. Ein falsch positives Ergebnis erscheint daher sehr unwahrscheinlich, allerdings kann eine Probenkontamination nicht vollständig ausgeschlossen werden. Die erneute Prüfung der prämortalen Abstrichproben war aufgrund fehlender Rückstellungen nicht möglich.

Zum aktuellen Zeitpunkt wurden über 50 Spender mittels postmortalem Nasopharynx‑/Konjunktivaabstrich getestet, bisher waren alle Ergebnisse negativ. Dies könnte aus der Tatsache resultieren, dass alle potenziellen Spender einer strengen Risikobewertung unterzogen werden und zudem die allgemeine Infektionsrate in der Spendenregion sehr gering ist. Im Netzwerk DGFG ist dies der einzige Fall, bei dem eine SARS-CoV-2-Infektion detektiert wurde.

Das Risiko der Virusinfektion über eine Gewebetransplantation ist bisher nicht einzuschätzen. Die Eignung vorhandener Tests für die postmortale Analyse ist unklar. Allerdings zeigten erste Untersuchungen an COVID-19-verstorbenen Patienten, dass SARS-CoV‑2 in Kornea und Konjunktiva bei einem Teil der Patienten nachgewiesen werden kann, wenn auch mit vergleichbar geringerer Virus-RNA-Konzentration [3]. In einer anderen Studie mit geringerer Fallzahl konnte SARS-CoV‑2 an okularen Geweben und intraokularen Flüssigkeiten nicht nachgewiesen werden [4]. Analysen zur Infektiosität von PCR-positiv getesteten Proben ergaben, dass die Virusanzucht aus Proben mit einem CT-Wert >24 und >8 Tage nach Symptombeginn nicht erfolgreich war [5]. Durch Analysen zur Korrelation von Viruslast und Anzüchtbarkeit der Viren in Zellkultur als Maß der Infektiosität lassen sich Grenzwerte ableiten, ab denen nur sehr geringe bis keine Infektiosität besteht. Das Robert Koch-Institut (RKI) hat empfohlen, dass ab einem CT-Wert >30 eine Kontrolluntersuchung zur Beurteilung weiterer Maßnahmen veranlasst wird [6]. Aktuell muss man davon ausgehen, dass eine Übertragung des Virus über transplantierte Gewebe wie Augenhornhaut potenziell möglich ist. Aufgrund der Anamneseerhebung fallen symptomatische Patienten als Gewebespender aus, sodass Patienten der präsymptomatischen Phase und symptomfreie Patienten als Risikogruppen verbleiben. Inwieweit diese Patienten ein Übertragungsrisiko darstellen, ist bisher unklar. Daher wäre es für den Empfängerschutz von Bedeutung zu wissen, ob ein Spender mit SARS-CoV‑2 infiziert ist.

Dieser Fall zeigt deutlich die diagnostische Lücke der verwendeten Testmethoden, da mit weitestgehend identischen Methoden das Virus 45 h nach dem ersten Test nicht mehr nachgewiesen werden konnte. Erste Hinweise deuten darauf hin, dass bei einer SARS-CoV-2-Infektion spezifische Antikörper erst nach der Serokonversion ab dem 7. bis 14. Tag nachgewiesen werden. Der IgA-positive, aber IgG-negative Serumbefund spricht für eine akute oder kürzlich durchgemachte Infektion mit SARS-CoV‑2. Der alleinige Antikörpernachweis ist daher nicht zur Akutdiagnostik empfohlen, sondern ergänzend zur Bestätigung eines positiven PCR-Abstrichtests. Die Verwendung von Antikörpertests sollte u. a. unter der Kenntnis der Spezifitäts- und Sensitivitätswerte des verwendeten Testsystems erfolgen [6]. Auch der verwendete Antikörpertest besitzt einen begrenzt positiven Vorhersagewert [7]. Über welchen Zeitraum SARS-CoV-2-spezifische IgM-, IgA- und IgG-Antikörper detektiert werden, ist bisher unklar, zumal im Nasen-Rachen-Raum trotz auskurierter COVID-19-Erkrankung weiterhin Virus-RNA nachweisbar sein kann. In Vergleichsuntersuchungen bei infizierten Patienten mit milden vs. schweren Symptomen wurden bei mild verlaufenden SARS-CoV-2-Infektionen erst 8 Tage nach Symptombeginn IgA-Antikörper nachgewiesen, wobei eine Korrelation zwischen der Intensität der Symptome und des Beginns des Nachweises bestand [8]. In Fällen mit schwachen Symptomen kann der Nachweis für IgG-Antikörper ausbleiben oder verzögert sein.

Der in dieser Arbeit vorgestellte Fall verdeutlicht folgende Fakten:

Im Fall der symptomarmen bzw. -freien Patientin zeigt sich deutlich die Wichtigkeit der routinemäßig durch die Kliniken durchgeführten Abstriche bei Aufnahme der Patienten, da der richtige Test zum richtigen Zeitpunkt durchgeführt werden muss [9]. Mit dem vorgestellten Fall kann keine Aussage darüber getroffen werden, ob der postmortale Abstrichtest für eine Prüfung auf SARS-CoV‑2 geeignet ist. Hierfür fehlt der valide Nachweis, dass mit dieser Methode postmortal eine Infektion mit SARS-CoV‑2 sicher festgestellt werden kann. Ob ein vorgeschlagener Test der entnommenen Augenhornhaut bzw. des Kulturmediums mittels qRT-PCR [10] für einen SARS-CoV‑2 Nachweis geeignet wäre, bedarf weiterer Analysen.

## Schlussfolgerungen

Nach der Serokonversion [9] könnte eine Analyse der Antikörper helfen, eine symptomarme Infektion zu erkennen und die diagnostische Lücke zu verringern. Die Sensitivität der ebenfalls getesteten Lateral-Flow-Assays ist den anderen Methoden, z. B. ELISA, deutlich unterlegen.

Ob eine Infektion mit SARS-CoV‑2 auch bei niedriger Viruslast bedeutet, dass Hornhautgewebe betroffen ist und ob eine Transplantation ein mögliches Infektionsrisiko darstellt, sollten Gegenstand weiterer Untersuchungen sein.

## References

[1] Paul-Ehrlich-Institut (2020) SARS-CoV-2: Wie sicher sind Gewebezubereitungen? https://www.pei.de/DE/newsroom/dossier/coronavirus/coronavirus-node.html. Zugegriffen: 24. Juni 2020

[2] Vogels CBF (2020). Analytical sensitivity and efficiency comparisons of SARS-COV-2 qRT-PCR primer-probe sets. medRxiv.

[3] Meinhardt J (2020). Olfactory transmucosal SARS-CoV-2 invasion as port of central nervous system entry in COVID-19 patients. bioRxiv.

[4] Bayyoud T (2020). Absence of severe acute respiratory syndrome-Coronavirus-2 RNA in human corneal tissues. Cornea.

[5] Bullard J (2020). Predicting infectious SARS-CoV-2 from diagnostic samples. Clin Infect Dis.

[6] Robert Koch Institut (2020) Hinweise zur Testung von Patienten auf Infektion mit dem neuartigen Coronavirus SARS-CoV‑2. https://www.rki.de/DE/Content/InfAZ/N/Neuartiges_Coronavirus/Vorl_Testung_nCoV.html. Zugegriffen: 11. Aug. 2020

[7] Caini S (2020). Meta-analysis of diagnostic performance of serological tests for SARS-CoV-2 antibodies up to 25 April 2020 and public health implications. Euro Surveill.

[8] Cervia C (2020). Systemic and mucosal antibody secretion specific to SARS-CoV-2 during mild versus severe COVID-19. bioRxiv.

[9] Wölfel R, Corman VM, Guggemos W (2020). Virological assessment of hospitalized patients with COVID-2019. Nature.

[10] Thaler S (2020). Bedeutung der Hornhautorgankultur bei Spendern mit möglicher SARS-CoV-2-Infektion. Ophthalmologe.

